# Residue-based multilayer-filters reused as amendment after capturing pig slurry gases

**DOI:** 10.1016/j.eti.2026.105111

**Published:** 2026-09

**Authors:** Adrián González-Guzmán, Vidal Barrón, Nadine Loick, Antonio Rafael Sánchez-Rodríguez, Alison Carswell

**Affiliations:** a Department of Agronomy, Rabanales Campus, University of Córdoba, Building C4, Córdoba PS:14071, Spain; b Net-zero & Resilient Farming, Rothamsted Research, North Wyke EX20 2SB, United Kingdom; c Department of Agronomy, Universidad de Sevilla, School of Agricultural Engineering (ETSIA), Seville PS: 41013, Spain

**Keywords:** Biofilters, circular economy, Nutrient cycling, Sustainable fertilizer, Waste recovery, Waste-based materials

## Abstract

Agriculture faces the challenge of reducing farm emissions and adopting circular economy strategies. This study assessed the formulation of three multilayer filters (MLFs) based on waste-derived materials from different industries. The study objectives were to (1) capture greenhouse gases and ammonia (NH_3_) and volatile sulfide compounds (VSC) from pig slurry and (2) assess the potential reuse of the most effective MLF as a soil amendment. Firstly, MLFs were designed and their efficacy to capture gases emitted from pig slurry (N_2_O, NO_2_, NH_3_, CH_4_ and CO_2_) evaluated. Secondly, MLF3 was used as a soil amendment (with and without previous exposure to pig slurry emissions) to evaluate its agronomic performance and environmental impact. For this, a soil was amended, introducing the variables sown (with *Lolium perenne*) or not-sown and with or without the nitrification inhibitor dicyandiamide. All MLFs captured > 75% of total N emitted from pig slurry and up to 95% of NO_2_ and NH_3_ emissions. The MLFs captured significant amounts of nitrogen, carbon and sulfur during the experiments, with maximum values of 4.4 g N kg^−1^ for MLF3 and 27 g C kg^−1^ and 2.5 g S kg^−1^ for MLF1. When MLF3 was amended to soil, the germination rate of *Lolium perenne* and root biomass increased*,* but above ground biomass was reduced. In addition, the loss of water soluble oxidized-N forms was reduced when soil was amended with MLF3. These results demonstrate the multifunctionality of MLFs in livestock manure management systems, as emission capture and nutrient recycling technology.

## Highlights

• Three multilayer filters (MLFs) were formulated using 12 waste-based materials.
• MLFs captured up to 95% of NH_3_ and NO_2_ from pig slurry.
• Reused MLF improved germination and root growth of *Lolium perenne*.
• N gas forms captured from slurry is then uptake by crop.
• Less water soluble oxidized-N forms were lost in soil amended with MLF3

## Graphical Abstract

**Figure:**
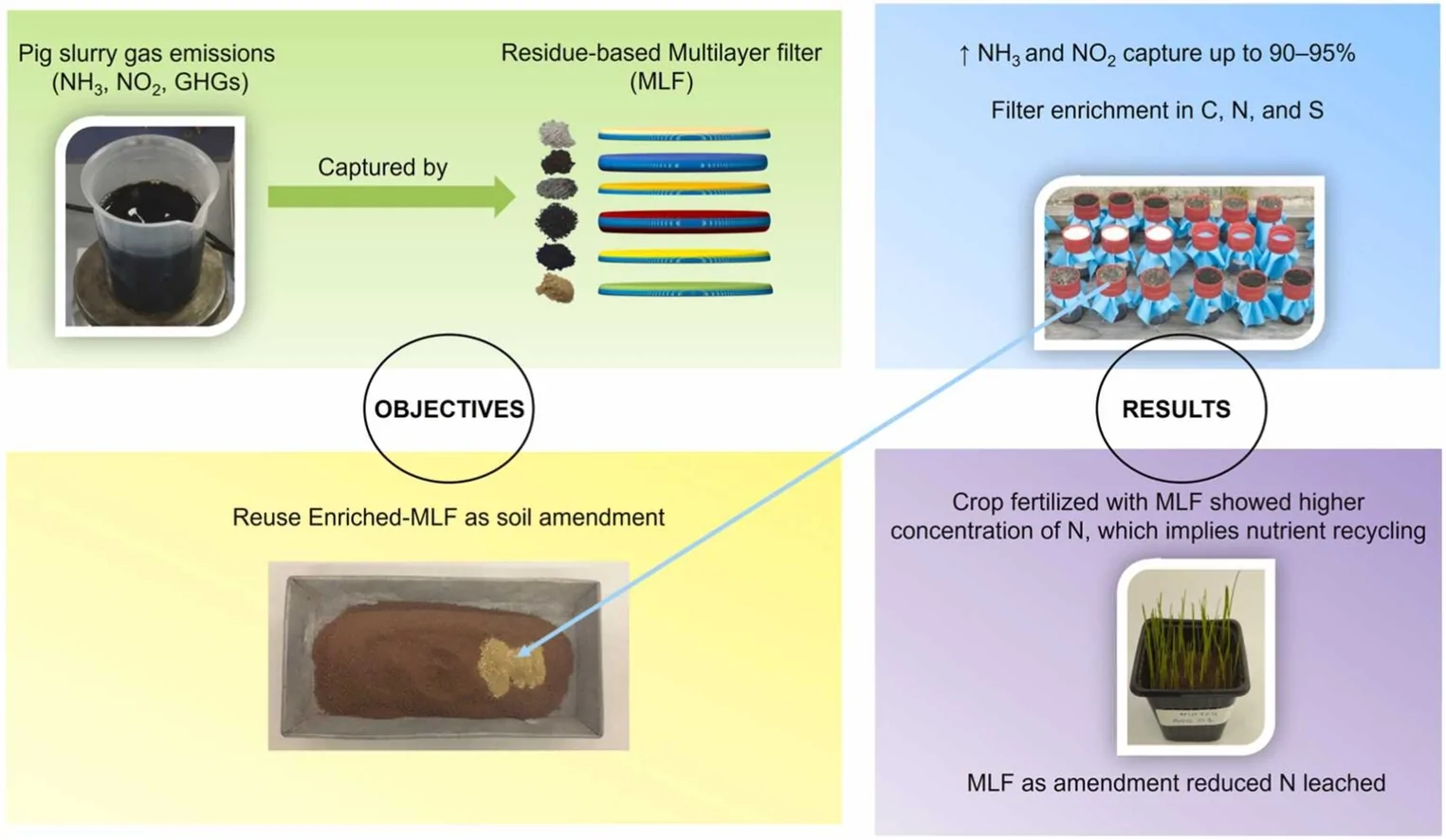

## 1 Introduction

In the 21st century agriculture faces the challenge of reducing greenhouse gas (GHG) and ammonia (NH_3_) emissions, particularly from livestock manure management, which represents a major emission hotspot (Bouwman et al., 1997, Richardson et al., 2023, Scheffler and Wiegmann, 2024). Swine production contributes 668 Mt CO_2_e year^−1^ (MacLeod et al., 2013)to global greenhouse gas emissions and ∼4.7 Mt year^−1^ NH_3_ to global NH_3_ emissions (Olivier et al., 1998, Uwizeye et al., 2020). To address this issue, regulatory frameworks promote emission mitigation strategies [Gothenburg Protocol and Best Available Techniques] in livestock systems such as dietary modifications, manure management practices and advanced technological methods. More recently, nitric oxide (NO) and nitrogen dioxide (NO_2_) emissions from slurry are also being studied because it has been shown that in the slurry headspace these gases can originate from microoxic nitrification processes, where ammonium (NH_4_) oxidation can produce NO and subsequently NO_2_ (Choi et al., 2023, Wang et al., 2021).

Gas purifiers from livestock farms —biofilters, scrubbers, or cleaners— are one of the technologies used to mitigate emissions of greenhouse gases (GHGs) and NH_3_ (Chen et al., 2009), with the first successful application and patent of biofilters registered in the 1950s (Leson and Winer, 1991). Efforts have predominantly focused on removing NH_3_ and odors caused by volatile organic compounds (VOCs), while other gases, such as CO_2_, CH_4_, N_2_O, and NO_2_, have received comparatively less attention (Van der Heyden et al., 2015). Despite their effectiveness, the adoption of these techniques is often hindered by high implementation costs, making them inaccessible to many farmers (Delgado et al., 2020). This highlights the need for cost-effective, scalable, and easy-to-implement alternatives that enable widespread adoption across farms, whilst ensuring environmental benefits.

In accordance with those social, economic and environmental needs, this study addresses three main challenges in agriculture, nutrient circularity, mitigation of GHGs, NH_3_ and volatile sulfide compounds (VSC) and synthesis of sustainable fertilizers, via an integrated approach. To reduce the environmental footprint of conventional agriculture, particularly around biogeochemical flows, there is an urgent need to reduce reliance on inefficient use of farm inputs, like synthetic or mined fertilizers, and extend the productive life of on-farm and other sector resources, in line with the approach of Blomsma and Brennan (2017). Europe generates millions of tons of residues per year from different industry sectors, representing an underutilized resource for circular economy strategies (Eurostat, 2024, INE, 2022). Therefore, low-value wastes need to be evaluated so alternative routes for their reuse can be found. The reuse of agro-industrial residues as soil amendments and fertilizers within the agri-food sector has emerged as a promising strategy (Chojnacka et al., 2020, European Commission. (2020)), however, these residues also have potential as biofilters (Van der Heyden et al., 2015). In this context, this study introduces a novel residue-based multilayer filter (MLF) system designed to simultaneously mitigate GHG and NH_3_ emissions from pig slurry and enable nutrient recycling via soil amendment. Compared with conventional biofilters, the proposed MLF technology represents a relevant conceptual advancement due to its structured multilayer configuration based on complementary functions rather than homogeneous mixed materials. Additionally, the combined use of inorganic and organic residues, and the predominance of physicochemical mechanisms such as adsorption and catalytic interactions, instead of relying mainly on microbial degradation, represents further developments in this area (Van der Heyden et al., 2015). In contrast to traditional biofilters, that generally target ammonia removal and generate exhausted materials requiring disposal or additional treatment, the MLFs are conceived within a circular economy framework. Thus, the main objectives of this study are (1) to evaluate the ability of a selection of waste-derived materials combined in an MLF to capture GHGsNH_3_ and VSC from a closed pig-slurry tank and (2) to assess their potential reuse as soil amendments enriched in N, C and S, in accordance with EU Regulation of Fertilizers 1009/2019, specifically CFP 3(b). We hypothesize that the designed MLFs are able to capture up to 90% of NH_3_ and more than 50% of other target gases emitted from pig slurry. Additionally, this nutrient enrichment of the residues can provide plant nutrition and will boost crop growth when applied as a soil amendment.

## 2 Materials and methods

### 2.1 Experimental design

A gas capture experiment with MLFs was conducted at the Campus of Rabanales (37°54'49.0"N 4°43'05.5"W), University of Córdoba, Spain (Experiment 1). This was performed under ambient external environmental conditions between March and April 2023. Temperature mean, maxima and minima were 17, 32 and 4ºC, respectively, whilst the mean relative humidity ranged from 63% to 38%, (see Figure S1). Four treatments and twelve experimental units were deployed (Figure S2A), comprising three replicates per MLF formulation (MLF1, MLF2, MLF3) and untreated control treatment. Each replicate included three filters (Figure S2B), and all were measured through a gas analyzer (Picarro G2508; Agilent Technologies Spain, S.L.) coupled with the chemiluminescence gas analyzer Acoem Serinus 40 (Agilent Technologies Spain, S.L.; Figure S2C and Figure S3).

Experiment 2 assessed the fertilization potential of MLF3 (original and enriched in C, S and N) and its potential to produce GHGs and NH_3_ following soil application, at Rothamsted Research, North Wyke. It was not possible to test all MLFs in this study because of the number of treatments and all measurements taken, including the use of a nitrification inhibitor, which was prioritized to elucidate its performance with this new amendment. In addition, MLF3 was selected for Experiment 2 because it had the greatest NH_3_ and NO_2_ mitigation potential. This second experiment was performed under controlled conditions of 12 h day and night and 25 and 21ºC day and nighttime temperatures, respectively (Figure S2D, S2E, S2F and S2G) between September and December 2023. The experimental design comprised three factors: (1) sown (*Lolium perenne*; perennial ryegrass) *vs.* not-sown soil; (2) original (i.e., MLF3 without enrichment) *vs.* enriched MLF3; (3) presence/absence of the nitrification inhibitor dicyandiamide (DCD). This 2 × 2 × 2 design, plus two controls (C: sown; C´: not-sown), yielded ten treatments with 4 replicates each (*n* = 40). The trial was conducted in two sequential runs (sown then not-sown soil), with six-week monitoring periods.

### 2.2 Characterization and composition of multilayer filters

Twelve waste-derived materials were selected from the collection of Research Group AGR−165 of the University of Cordoba to be used as layers for the MLFs (Table 1). The waste-derived materials (hereafter “residue/s”) were collected from different companies from the south of the Iberian Peninsula, related to agriculture, industry, domestic, and forestry sectors. The nature of materials is either organic or inorganic, with substantial differences in their physicochemical properties (Table S1).

**Table 1:** Description of the twelve residues used in MLFs, detailing the MLFs formulations (mass proportion of each residue) and arrangement of each residue within it, being number one the layer closest to the slurry.

**Residues**	**Description**	**MLF1**		**MLF2**		**MLF3**	
		**%**	⇅	**%**	⇅	**%**	⇅
Al chips	Fine aluminum shavings and dust from cutting and machine use	12	9				
Al sludge	Residual sludge from the aluminum extrusion process					12	2
Bentonitic sands	Waste from the steel industry, used in mold casting due to its binding and thermal resistance properties			10	6		
Black slag biomass	Mix of bed slag from furnace for energy production and uncombusted biomass			5	5		
Compost EM	Composted sludge, stabilized organic waste from wastewater treatment, often used as a soil amendment or fertilizer	10	4	15	3		
Cork	Cork bark waste from the cutting process, consisting of residual fragments and shavings	14	3	20	2		
Iron chips	Fine residual dust from laser cutting of iron sheets	4	2				
Iron slag	Waste from the steel industry, generated in the basic oxygen furnace (BOF) process	2	8				
Olive ash Baena	Olive pit combustion residue, rich in minerals, and sometimes used as a soil amendment	5	7				
Orange peel	Residual byproduct from orange processing, rich in organic matter and fiber	20	1	40	1	88	1
Sludge M	Municipal wastewater treatment sludge, composed of organic and inorganic matter	20	6				
UBCEP^2^	Residual organic material from cellulose production, consisting of unprocessed biomass fibers and plant matter rich in sodium	13	5	10	4		

^1^Al: Aluminum; ^2^UBCEP: Uncooked biomass from the cellulose extraction process; ⇅ means filter layer arrangement with air passing through the filter from layer 1–9, 6 or 2, respectively.

The filter design approach moves beyond single-material biofilters towards engineered multi-functional systems, with the MLFs formulation designed to address two criteria: (1) the capability of the individual residues to capture gases, and (2) the nutrient properties of the residues. The residue capability to capture N was estimated in a previous study (González-Guzmán et al., 2026). These previous results led us to select specific residues to reach a maximum, and balanced, gas mitigation among all target gases emitted from slurry. The formulation also met the physicochemical properties of EU Regulation 2019/1009 for fertilizers specification, because it is mandatory for its reuse as fertilizer after saturation. The main criteria for selecting the order of the filter layers were to use the residues that captured the greatest amount of N (e.g. orange peel and cork) as the first layer that made contact with slurry gases to avoid any subsequent transformation through the filter, for example via nitrification or denitrification. The last layer was Al chips and Al sludge because of their photocatalytic properties. Iron oxide, for example, was placed before a layer that captured CO_2_ (Olive ash Baena) because it seemingly showed CH_4_ transformation to CO_2_. However, other layers within the filter were placed to increase the capture of those or other gases, but also to confer the adequate properties for being used as a soil amendment. Thus, the physicochemical properties of the MLFs were estimated as follows: First, the dry matter of the residue (*DMR*) was measured through volume (*V*), density (*ρ*) and moisture (*θ*); second the proportion of residue *x* in the materials combination [*DMR*_*rx*_ *(%)*] was obtained by dividing the dry matter content of residue *x* (*DMR*_*rx*_) between the total dry matter weight of all residues used (*ΣDMR*_*rx-ry*_). Subsequently, the physicochemical properties of the final MLFs such as total N concentration (*TN*_*MLF*_) were estimated by the sum of the product and of the proportion (in dry weight) of each residue in the filter, $\sum_{\mathrm{rx}}^{\mathrm{ry}}\left(\mathrm{DMR}\right)$ multiplied by the concentration of the chemical elements contained in it (*TN*) ((1), (2), (3), respectively):

(1) DMR=ρ*V*(1−θg)

(2) $${\mathrm{DMR}}_{\mathrm{rx}}(\%)=\frac{{\mathrm{DMR}}_{\mathrm{rx}}}{{\Sigma \mathrm{DMR}}_{\mathrm{rx}-\mathrm{ry}}}\times 100\;$$

(3) $${\mathrm{TN}}_{\mathrm{MLF}}=\;\sum_{\mathrm{rx}}^{\mathrm{ry}}\left(\mathrm{DMR}\right)*\left(\mathrm{TN}\right)$$.

The formulation procedure was the same for the rest of the nutrients and potential pollutants. The MLFs evaluated here were engineered materials and should not be considered as raw industrial wastes. Their formulation follows a defined manufacturing protocol, and their potential agronomic reuse must comply with national and international regulatory frameworks governing fertilizing products, including EU Regulation of Fertilizer 1009/2019, specifically for CFP 3(b) inorganic amendment.

### 2.3 Physicochemical analysis of residues

Composite samples of each residue were transported to the laboratory, where they were air-dried for 72 h. Following which all materials were crushed and sieved to pass through a 2-mm mesh (except UBCP which developed a cotton-like fibrous texture, making size reduction impractical and we used scissors). The physicochemical analyses were performed on the < 2 mm fraction of each material. Texture was assessed using a vibrating sieve tower (ø mm: 2 – 0.5, 0.5 – 0.2, 0.2 – 0.05, and < 0.05); specific surface area (SSA) was determined as described by Tuller and Or (2005), additionally, density (ρ), electrical conductivity (EC, 1:5 SMEWW 2017a), and pH (1:2.5, *m*:*V* SMEWW 2017b) were measured. Total C, N, and S (C_t_, N_t_, and S_t_) were analyzed using a LECO CN628 analyzer (LECO Instrumentos S.L.; using LECO Corporation (1999) method), while organic C (C_o_) was quantified via the Walkley, Black (1934) method. Labile organic C (C_p_) was extracted using sodium pyrophosphate (Bascomb, 1968). Available P was determined following Olsen et al. (1954). Nitrate (NO_3_) and ammonium (NH_4_) concentrations were quantified spectrophotometrically using a Bio Tek Power-Wave-XS microplate reader (Agilent Technologies Spain, S.L.), applying the methodologies of Miranda et al. (2001) and Mulvaney (1996), respectively.

Cation exchange capacity (CEC) and both exchangeable acid and non-acid cations were extracted with 1 M NH_4_OAc at pH 7 and quantified via Flame Atomic Absorption Spectroscopy (F-AAS, Perkin Elmer). The available fractions of Fe, Cu, Mn, and Zn were extracted using the diethylenetriamine pentaacetic (DTPA) method (Lindsay and Norvell, 1978) and also measured using F-AAS (Fe_DTPA_, Cu_DTPA_, Mn_DTPA_, Zn_DTPA_). The water-soluble fraction of chemical elements (Ag, Al, As, Ba, Ca, Cd, Co, Cr, Cu, Fe, Hg, K, Mg, Mn, Mo, Na, Ni, P, Pb, S, Sb, Se, Si, Sn, Sr, Tl, Ti, V, and Zn) was extracted by mixing the residue with water (1:20 m:V) for five days, following Buján et al. (2010a) and analyzed using Inductively Coupled Plasma Optical Emission Spectroscopy (ICP-OES, Agilent Technologies Spain, SL). The total concentrations of these elements were determined via acid digestion with concentrated HNO_3_, HCl, and 30% H_2_O_2_ (EPA, 1996), followed by ICP-OES analysis. The swine slurry sample (30 L) was collected from a farm pit in the west of Lisbon, Portugal, and its TN content was quantified using the Kjeldahl method (N_k_). It should be noted that physicochemical analysis of either residues, soils or plant material was done in different laboratories (AGQ Labs, ActLabs, Researching Support Central Service of the UCO and the own laboratory of the AGR−165 Researching Group of UCO). Analytical quality was ensured using laboratory-specific quality control procedures. AGQ performed the analyses following internal methods based on ISO/TS 16965 and ISO 11466:1995 coupled with EN ISO 17294–2:2017, depending on the analyte. Results below or above the validated quantification range were denoted by the symbols "< " and "> ", respectively. Analytical recoveries ranged from 90 to 105% for organic materials and 85–105% for inorganic materials. Quality control was verified using a custom-made SPC Science multi-element standard (Catalogue No. AQ0–121–101) at SCAI, the certified reference materials NIST SRM 1515 (Apple Leaves) and NIST SRM 1547 (Peach Leaves) at AGQ, and the European Commission Joint Research Centre reference material Sediment S25 together with INRA 33 Poudre de Maïs (BIPEA) at the University of Córdoba laboratory. The limits of quantification can be seen in Table S2.

### 2.4 Experiment 1: Gas capture efficacy of multilayer filters

After adjusting the three MLF formulations, experimental units were built. Each experimental unit contained three technical replicates. The technical replicate was formed using a beaker filled with pig slurry (20 g slurry in a 50 mL beaker), covered with a cloth and the cloth secured by a screw top lid (Figure S2B). The lid was modified to be filled with each residue layer (Table 1), making a total of 3 × 8 g (dry weight) filter units for each experimental unit of each treatment. The twelve experimental units were placed inside the twelve automatic chambers (46 L volume) and randomly distributed (Figure S2A and S2B). Gas emissions were monitored using a static, non–steady-state chamber system, in which no force was applied and gas transfer occurred by passive diffusion through the filters. Each experimental unit was placed inside an automatic chamber, and they were sequentially connected to a multiplexer system, which controlled chambers closure and air sampling. When a chamber was closed, gas concentrations accumulated in the headspace were measured for 15 min using two coupled analyzers: a Picarro G2508 (NH_3_, N_2_O, CH_4_, CO_2_ and H_2_O) and a chemiluminescence analyzer (NO and NO_2_). Air was continuously drawn from the chamber at a combined flow rate of 0.9 L min⁻¹ . (Picarro G2508 was 0.35 L min^−1^ and the Acoem chemiluminescence analyzer was 0.55 L min^−1^). After each measurement period, chambers were opened and flushed with ambient air for 15 min before the next chamber was sampled. This sequential measurement cycle allowed all 12 chambers to be monitored within a 6-hour period, with four measurements per chamber per day (1440 min day^−1^ / 12 chambers / 30 min sampling and flushing period). To minimize the effects of air disturbance associated with chamber opening and closing, data from the first and last 60 s of each measurement period were excluded from flux calculations. This exclusion period was also necessary to avoid measuring residual gas remaining in the sampling lines (approximately 40 s residence time), ensuring that measurements reflected the actual chamber atmosphere. This approach also improved the linearity of gas concentration changes while maximizing the available measurement period. This was done modifying the initial minute and focus on NH_3_ due to it is the gas that presented the higher delay due to its stickiness. A total of 88 cycles were performed over 23 days after which the slurry in all treatments had dried out. However, emissions dropped significantly (∼90%) after cycle 40 (day 10) in all treatments. Therefore, only data from the first 13 days was considered when investigating the efficacy of the different MLFs. Environmental temperature was recorded throughout the experiment to account for its influence when calculating gas fluxes. Total N of the starting slurry (N_k_) and final dry slurry (N_t_) was used to calculate how much N was volatilized and passed through the MLFs. After 23 days MLF material was removed and stored in plastic bags at room temperature for 4 months, after which MLF3 was transferred to Rothamsted Research for use in Experiment 2.

#### 2.4.1 Determination of nitrogen, carbon and sulfur enrichment of multilayer filters

Retention of N, S and C in the MLF was used as a proxy of gas capture efficacy and enrichment (MLF-E_N/C/S_). The MLF-E_N/C/S_ was calculated using the initial MLF C_t_, N_t_, and S_t_ concentration (see Eq. 3; C_i_, N_i_ and _Si_, respectively) and the final MLF concentrations (MLF-E, C_f_, N_f_ and S_f_), as follows:

(4) $${\mathrm{MLF}-E}_{X}(\%)=\frac{\left(\mathrm{Xf}-\mathrm{Xi}\right)}{\mathrm{Xi}}\times 100$$.

*Being X: C, N or S.

To compare the efficacy among filters we estimated the total N volatilized by subtracting the N contained in the dry matter slurry (DMR) from the total N in the liquid slurry (LS).

#### 2.4.2 Capture efficacy by flux calculation method

This method calculates gas fluxes in g m^−2^ h^−1^ by linear regression (LR). This is calculated by the variation of the concentration of the gas (NH_3_, CO_2_, CH_4_, N_2_O and NO_2_) in a specific range of time (slope), multiplied by the volume of the chamber and divided by the surface emission of the pig slurry (Maier et al., 2022):

(5) $$F\;=\frac{\mathrm{dC}}{\mathrm{dt}}\times \frac{V}{A}$$.

Atmospheric pressure was considered constant, temperature was monitored at 10 min frequency and was used to calculate the flux and the increasing concentration of gas was considered linear where the R_2_ was > 0.6 (established based on author´s experience to better adjust linearity of all gases along the whole experiment). The final capture efficacy was calculated according to Eq. 6, where ‘*F*_*C*_‘ is the control total emission mean per day and ‘*F*_*MLF*_‘ is the MLF total emission for each replicate of each treatment per day.

(6) $$\mathrm{Efficacy}\;\left(\%\right)=\frac{{(F}_{C}-\;F_{\mathrm{MLF}})}{F_{C}}\times 100$$.

#### 2.4.3 Capture efficacy by the area under the curve method

Since there were data that did not meet our linear regression criteria (R^2^ < 0.6), mainly CH_4_ and N_2_O, net flux (F, g m^−2^ h^−1^) for all gases (NH_3_, CO_2_, CH_4_, N_2_O and NO_2_) was also calculated using area under the curve (AUC) as explained in Barrón et al. (2020). This consists of the difference between the sum of the concentration of each gas in each chamber per time (ppm × min) and the sum of the atmospheric concentration for that gas $\left(\sum_{2}^{14}C_{\mathrm{sample}-}C_{\mathrm{atm}}\right)$ within the 13 min measurement window (Time, 15 min minus first and last minute). Then, it was divided by the number of minutes measured and multiplied by 60 to obtain concentration (ppm) per hour. After that, the result is divided by emission surface (m^2^) to finally convert volume concentration (L) into mass (g) through ideal gas law, where just the T (temperature) was adjusted according to the data recorded, and finally, MW is the molecular weight of the gas measured.

(7) $$F\;=\frac{0.9L}{\min}\times \frac{\left(\sum_{2}^{14}C_{\mathrm{sample}-}C_{\mathrm{atm}}\right)}{\mathrm{Time}\;(\min)}\times 60\times \;\frac{1}{A}\times \frac{P}{R\times T}\times \mathrm{MW}$$.

The flow rate of the analyzers was also considered (0.9 L min^−1^, 0.35 L from Picarro G2580 and 0.55 L from chemiluminescence analyzer), allowing daily cumulative gas emission (mg m^−2^) to be calculated.

### 2.5 Experiment 2: Gas emission and agronomic potential of MLF3 as a soil amendment

The second experiment used MLF3 as an inorganic amendment to sown and not-sown soil. GHG and NH_3_ emissions were then monitored after the MLF3 amendment. Three factors were assessed as summarized in Table 2.

**Table 2:** Description of the five treatments (Soil is the control treatment, MLF3 and MLF3-NI are the original multilayer filters without or with nitrification inhibitor^a^, respectively; MLF3-E and MLF3-E-NI are enriched multilayer filters without or with nitrification inhibitor, respectively) and the factors tested (enriched, nitrification inhibitor) either in *not-sown* and *sown* soils. Absence of a factor is indicated by "0" and presence by "1".

		**Soil**	**MLF3**	**MLF3-NI**	**MLF3-E**	**MLF3-E-NI**
**Not-Sown**						
	**N-Enriched**	0	0	0	1	1
	**NI**	0	0	1	0	1
						
**Sown**						
	**N-Enriched**	0	0	0	1	1
	**NI**	0	0	1	0	1

a NI: Nitrification inhibitor dicyandiamide (DCD).

#### 2.5.1 Experiment 2 settings

The soil used was a Dystric Cambisol (Harrod and Hogan, 2008, IUSS Working Group WRB, 2014), which was under permanent pasture, collected from the North Wyke research site. This soil was air dried and passed through a 2 mm sieve prior to use. Each pot (5 × 5 × 6 cm) was base sealed, filled with 150 g dry soil and packed to a bulk density of 1.16 kg dm^−3^, using a total of 6 kg soil (10 treatments × 4 replicates × 0.15 kg). Soil was kept at 60% water filled pore space (WFPS) to optimize conditions for nitrification (Linn and Doran, 1984), using potable water. The amount of water (mL) needed to keep soil at 60% of WFPS was calculated according to this formula:

(8) $$V=\frac{\mathrm{WFPS}}{100}\times \;V_{s}\times (1-\frac{D_{b}}{D_{p}}\;)$$

Where V is the mL of water that must be added to the soil to maintain 60% WFPS; $V_{s}\;$is the volume of the 150 g of air dried soil; $D_{b}$ is the bulk density (1.16 g cm^−3^) and $D_{p}$ is the particle density (2.65 g cm^−3^). Soil was maintained at 60% WFPS by weighing the pots every two days to replace lost moisture via evaporation and transpiration.

The inorganic soil amendment used in this experiment was MLF3 from Experiment 1, either as original (MLF3, without being used as filter) or enriched (MLF3-E), to evaluate the effect of transferring the nutrients captured from slurry to soil. A total of 1.8 g of MLF3 or MLF3-N was applied to each pot simulating an application of ∼7.2 t ha^−1^, which equates to an application of 77 and 100 kg of N per ha. The MLF was mixed with the soil before filling the pots, and the nitrification inhibitor dicyandiamide (DCD) was applied with the first soil watering. The dose of DCD chosen was the highest allowed by the European Union (EU) legislation, at 40 g per kg of N applied, which led to application rates of 0.80 and 1.04 mg per pot, for original and enriched MLF3, respectively.

Pots were sown with 0.3 g of seeds (136 ± 3 seeds) of *Lolium perenne* (L.). This intentionally high density was selected to ensure full root exploration of the soil volume, thereby accelerating the mineralization of the nutrient-enriched MLF3 material. This setup was designed as a mechanistic test of nutrient release and potential gaseous N losses to compare with not-sown soils, rather than to simulate agronomic field sowing rates. Plants were grown in a controlled environment room (CER) programmed 12/12 h light/dark and air moisture 70 ± 5%. Ten days after sowing (DAS) plants were counted and six weeks after sowing, the plants were cut to one cm above soil surface, weighed, and washed with potable water, before drying them at 55 ºC for 5 days.

Once plants were dried, aerial dry matter (ADM) was recorded and total N, C and S concentration was measured. The dried plant material was ground and digested in HNO_3_ for elemental analysis. Root dry matter (RDM) was measured per pot following the same methodology and total dry matter (TDM) was calculated by the sum of both (ADM and RDM). The setting up of unsown pots was the same but without seeds sown.

#### 2.5.2 Gas emission measurements

Greenhouse gas and NH_3_ emissions were monitored for 25 days in sown or not-sown soils. Throughout this time two coupled measurements were performed. Firstly, NH_3_ was measured by dynamic throughflow chambers with acid traps (H_3_PO_4_ 0.1 N) installed at the outlets (Figure. 2SC, 2 SD and 2SE), these were running during the dark period (12 h), following the method of (Drame et al., 2023). The system consisted of 20 sealed cylinders where pots were kept during the night and an extra 2 cylinders with no pot inside to measure baseline NH_3_. Flow was generated using an electrical pump and regulated by individual flow meters, maintained at 2 L min^−1^. The trapped NH_3_ was measured using discrete photometric analysis (Krom, 1980, Searle, 1984). Secondly, Licor (LI−8100A Automated Soil CO_2_ Flux System) and Gasmet (GT5000 Terra) instruments were linked to a multiplexer (LI−8150), that in turn was connected to three automatic chambers (8100–104 C) used to measure CO_2_, CH_4_, N_2_O and NH_3_ (Figure. 2SF) during the light period. The measurement sequence time used during the day was 3 min of purging, followed by 16 min measurement period, ending with a 1 min purge. Thus, each sample measurement period lasted 20 min, and all samples (the 20 pots) were measured within 7 h. Pots were changed manually every hour, starting from replicates 1 of each treatment (Soil>MLF3 > MLF3-NI>MLF3-E > MLF3-E-NI) and finishing with replicate 4, having the same time between measurements.

#### 2.5.3 Forms of nitrogen in leachate

Water soluble N was extracted from the not-sown soil by saturating the soil beyond 100% WFPS at the end of the experimental period. After the seal was removed from the bottom of the pot, water was added to the pots via the soil surface at ∼ 2 mL min^−1^ for over 1 h. The soil was then allowed to freely drain for 3 h, and the leachate collected. Ammonium-N (NH_4_-N), total oxidized nitrogen (TON), and TN were measured colorimetrically (Aquakem 250 analyser, Thermo Scientific) and a modified Berthelot reaction involving salicylate and sodium dichloroisocyanurate was used to measure NH_4_-N (Krom, 1980, Searle, 1984). Total N samples were digested and subsequently analyzed for TON using a cadmium reduction step (for nitrate to nitrite) followed by a diazotization reaction and spectrophotometric detection.

### 2.6 Statistical analyses

Statistical analyses and graphical visualizations of gas emissions from MLF treatments were performed using the software Statistix 10 v.10.0, Sigma Plot 11.0 and RStudio software (version 4; R Core Team, 2024). The following R packages were employed: *dplyr* for data manipulation, *ggplot2* for graphical representation, *agricolae* for post-hoc analysis using the Least Significant Difference (LSD) test. The gas emissions from soil were processed using Soil Flux Pro 5.3.0 software (LI-COR Biosciences Inc., 2023). Gas fluxes and variables such as germination (10 DAS), ADM, RDM, TDM; nutrient uptake, cumulative NH_3_ emissions and the different N fractions leached were statistically analyzed with the software Statistix 10 (v 10.0). Data was analyzed using ANOVA with a completely randomized design to explore differences between the five treatments. After that, an LSD test was applied post-hoc, where significant differences were observed. Lastly, to study the interactions of the factors applied in this study, a factorial ANOVA analysis was performed —omitting the Control treatment— and using original (MLF) or enriched MLF (MLF-E), and with and without NI as factors. In this work differences were considered significant where *p* < 0.05.

## 3 Results

### 3.1 Physicochemical properties of soil and multilayer filters

The physicochemical properties of the MLFs formulated in Experiment 1 and of the soil used in Experiment 2 are presented in Table 3. MLFs possess a high specific surface area, which is key for effective gas capture, and they have a high N and C content, which can contribute to soil fertility. Another aspect that ought to be considered is the low ratio of plant-available/total elements.

**Table 3:** Soil (Dystric Cambisol) and multilayer filter (MLF1, MLF2 and MLF3) physicochemical properties.

**Parameter**		**Units**	**Soil**	**MLF1**	**MLF2**	**MLF3**
**Particle size**	**Coarse Sand**	%	43	60	45	27
	**Fine Sand**			24	32	33
	**Silt**		37	16	23	40
	**Clay**		20			
	**SSA**^**1**^	m^2^ g^−1^	NA	327	419	573
	**pH**		5.8	NA	NA	NA
	**CE**	(µS cm^−1^)	NA	6164	7758	4749
**Cation Exchange Complex**	**Ca**	cmol_c_ kg^−1^	NA	59.2	57.1	14.1
	**Mg**		NA	8.6	5.2	2.7
	**Na**		NA	11.8	16.4	10.4
	**K**		NA	39.0	9.5	10.5
	**CEC**^**2**^		10	119	88	38
	**P**_**Olsen**_	mg kg^−1^	19.3	83.5	83.0	73.3
	**N**_**t**_^**3**^	g kg^−1^	0.19	13.47	10.43	11.22
	**C**_**p**_^**4**^		NA	4.05	3.12	0.00
	**C**_**o**_^**5**^		NA	215	257	297
	**C**_**t**_^**6**^		2.4	288	321	385
^1^Specific Surface Area;^2^ Cation exchange capacity;^3^ Total nitrogen;^4^ carbon extracted with Na-pyrophosphate;^5^Organic Carbon;^6^Total Carbon						

Soil and multilayer filters (MLF) available and total fraction of chemical elements							
**Element**	**Soil**	**MLF1**		**MLF2**		**MLF3**	
	Available	Available	Total	Available	Total	Available	Total
**Al**	900	< 1	17400	< 1	10100	< 1	9600
**As**	0.19	< 0,3	6	< 0,3	6	< 0,3	4
**B**	0.34	< 0,02	45	< 0,02	16	< 0,02	49
**Ba**		< 0,2	380	< 0,2	127	< 0,2	21
**Ca**	1500	1800	23700	1500	15900	1300	34400
**Cd**	0.08	< 0,02	0	< 0,02	0	< 0,02	0
**Cl**	NA	5	1100	4	600	< 0,01	48
**Co**	0.54	< 0,02	5	< 0,02	1	< 0,02	0
**Cr**	0.15	< 0,2	282	< 0,2	15	< 0,2	37
**Cu**	5.2	2.9	512.3	1.3	30.8	0.4	6.1
**Fe**	383	21	35000	< 0,02	6400	17	1200
**K**	236	6600	9900	2700	9300	5000	7800
**Mg**	73	421	7000	393	4150	448	1750
**Mn**	166	5	832	5	303	1	14
**Mo**	0.03	< 0,05	10.72	< 0,05	0.41	< 0,05	1.43
**Na**	23	976	2500	446	2800	376	2100
**Ni**	0.9	< 0,05	160	< 0,05	12	< 0,05	20
**P**	57	168	5900	301	2300	609	800
**Pb**	7.5	< 0,1	614	< 0,1	10.3	< 0,1	0.7
**S**	10	33	3381	25	1090	0	1187
**Sb**	NA	< 0,1	20	< 0,1	0	< 0,1	0
**Si**	NA	67	10800	34	24800	16	288
**Sn**	NA	< 0,1	67	< 0,1	2	< 0,1	13
**Sr**	NA	7	190	8	69	13	34
**Ti**	0.5	< 0,1	12700	< 0,1	357	< 0,1	372
**Zn**	11.0	2.8	632.9	2.3	92.2	3.1	13.3

Available elements were extracted using the Mehlich III method (Mehlich, 1984) for soil and deionized water (1:20 m:V) (Buján et al., 2010b) for MLFs.

### 3.2 Enrichment and efficacy of multilayer filters

A significant increase in C, S and N in all MLF assessed was found after their use as filters. Carbon content increased by 26.9, 18.2 and 4.4 g kg^−1^ in MLF1, MLF2 and MLF3 (*p* = 0.002, 0.199 and 0.703 respectively), compared to their former contents before the experiments, whilst S increased by 2.5, 0.7 and 1.2 g kg^−1^ (*p* = < 0.001 for all MLFs). The N balance (at the end of the experiment) (Figure S4) demonstrates that both the total N in the DMS (∼18 mg) and the N volatilized (∼110 mg) were similar in the four treatments, whilst the N captured by MLFs ranged between 98% and 76% of MLF1 and MLF3, respectively, being significantly different MLF1 relative to MLF2 and MLF3.

**Fig. 1:**
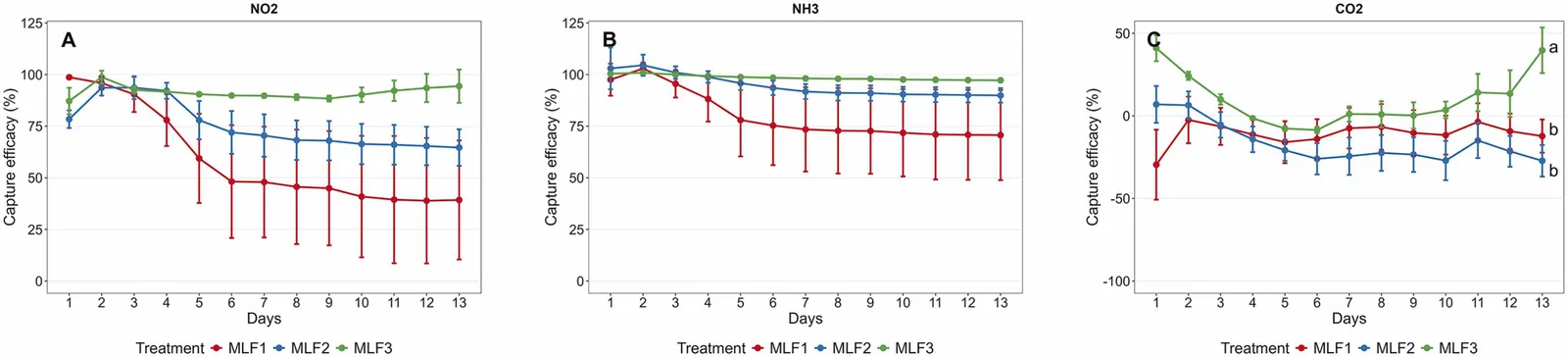
Capture efficacy (%) of NO_2_-N, NH_3_-N and CO_2_-C gases for each of the three multilayer filters (MLF) relative to the Control treatment. Data obtained with linear regression (LR) method. Different letters between treatments indicate significant differences (*p* < 0.05) after one-way ANOVA (*n* = 3). Abse*n*ce of letters means no statistical differences.

Fig. 1 shows the efficacy of each MLF relative to the control treatment for NO_2_, NH_3_ and CO_2_ determined by LR (Figs. 1A, 1B and 1C, respectively), whilst Figure S5 shows the same results calculated by AUC. Figure S6 showed the same results for CH_4_ and N_2_O. Capture efficacy and gas speciation results obtained through linear regression (LR) showed higher accuracy than area under the curve (AUC) method in terms of absolute values (one order of magnitude inferior). Therefore, even though relative values were very similar (Figure S7 and S8), the LR method was deemed most appropriate for reporting here. The NO_2_ and NH_3_ capture efficacy was higher with all MLFs during the 13-day monitoring period, except MLF1, which showed a decrease after the fifth day (Fig. 1 A, 1B, S5A and S5B). In contrast, CO_2_ capture efficacy had more variation between methods and a positive net emission (Capture efficacy < 0%) (Fig. 1 C and S5C). Both methods showed statistically significant differences for NO_2_ emissions between MLF2 and MLF3 relative to the Control (Figure S7) and MLF3 tended to have lower NO_2_ emissions than MLF1, although they were not statistically different (Fig. 1, *p* value = 0.059). For NH_3,_ all MLFs showed significant emission reduction than Control (Figure S7). Statistically higher CO_2_ capture efficacy with MLF3 respect to other treatments was observed (Figure. 1 and S7) but higher emission of N_2_O compared to MLF1 (Figure S8). Finally, MLF2 showed the highest capture of CH_4_, being significantly different respect MLF3 (Figure S6).

### 3.3 *Lolium perenne* performance

#### 3.3.1 Seed germination and biomass yield

**Fig. 2:**
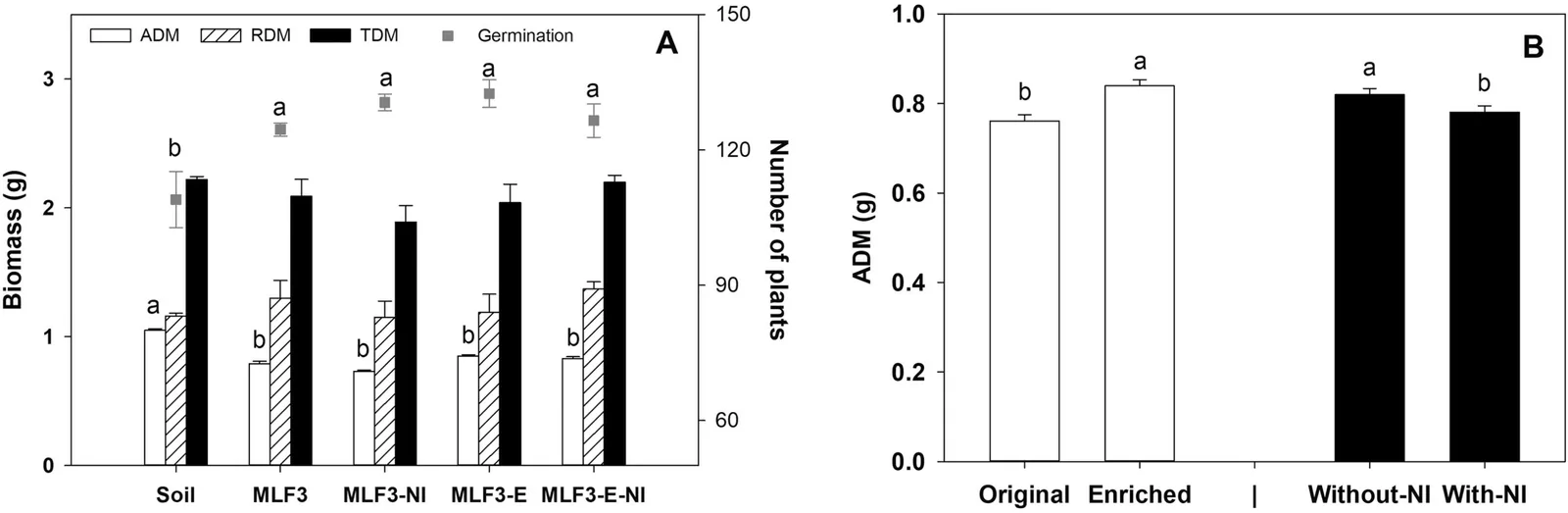
Panel A presents plant biomass [aerial dry matter (ADM), root dry matter (RDM) and total dry matter (TDM)] and number of ryegrass plants germinated for the five treatments (“Soil” is the Control, MLF3 and MLF3-NI are the original multilayer filters without or with the nitrification inhibitor dicyandiamide, respectively; MLF3-E and MLF3-E-NI are multilayer filters enriched with the emitted pig slurry gases, without or with nitrification inhibitor, respectively). Bars are mean values ± SE (*n* = 4). Panel B presents ADM result from the factorial ANOVA, using enrichment and nitrification inhibitor as factors. Bars are mean values ± SE (*n* = 8). Different letters above bars indicate significant differences (*p* < 0.05) after one-way ANOVA or Factorial ANOVA test, respectively.

Application of MLF3 to soil significantly increased germination rate (*p* = 0.003) and decreased ADM (Fig. 2A). However, when TDM of all treatments were compared they were statistically similar (Fig. 2A). The factorial ANOVA analysis showed no significant treatment interaction for ADM, so ADM results were grouped as original and enriched MLF and with and without NI (Fig. 2B). Enriched MLF was found to produce significantly greater ADM than original MLF, and ADM was significantly reduced when NI was included (Fig. 2B).

#### 3.3.2 Nutrient uptake

**Fig. 3:**
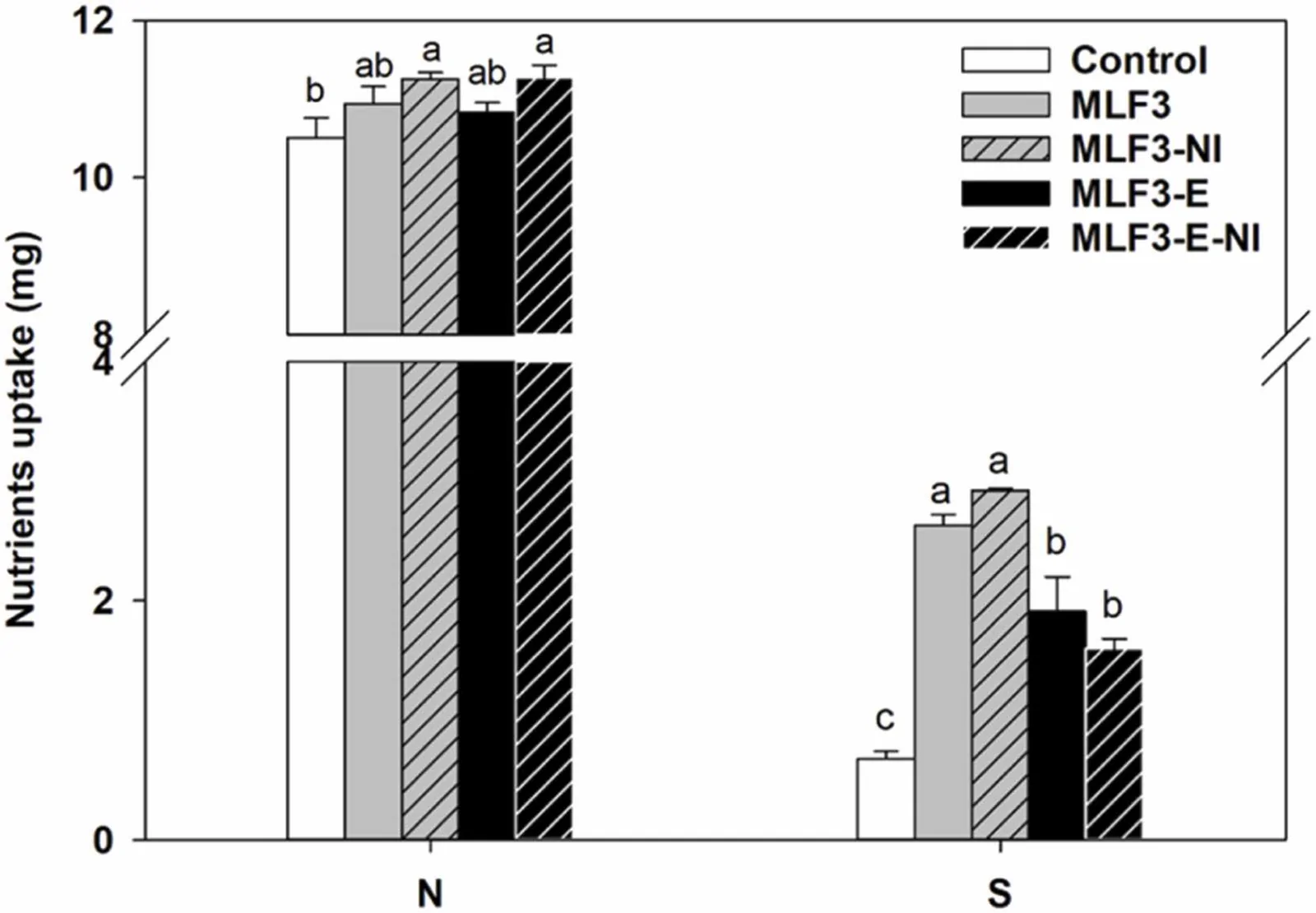
Nitrogen (N) and sulphur (S) uptake by *L. perenne* under the different experimental treatments where “Soil” is the control treatment, MLF3 and MLF3-NI are the original multilayer filters without or with the nitrification inhibitor dicyandiamide treatments, respectively; MLF3-E and MLF3-E-NI are multilayer filters, enriched with emitted pig slurry gases, without or with the nitrification inhibitor treatment, respectively. Bars are mean values ± SE (*n* = 4). Different letters between bars indicate significant differences (*p* < 0.05) after one-way ANOVA analysis.

Nitrogen uptake by *Lolium perenne* sp was significantly greater for the treatments where NI was included, relative to the Control (*p* = 0.047). Sulphur uptake was significantly higher in all MLF treatments relative to the Control (Fig. 3). Further, S uptake was significantly greater in the original MLFs than the enriched MLF treatments (Fig. 3; *p* < 0.001).

### 3.4 Soil gas emissions

**Fig. 4:**
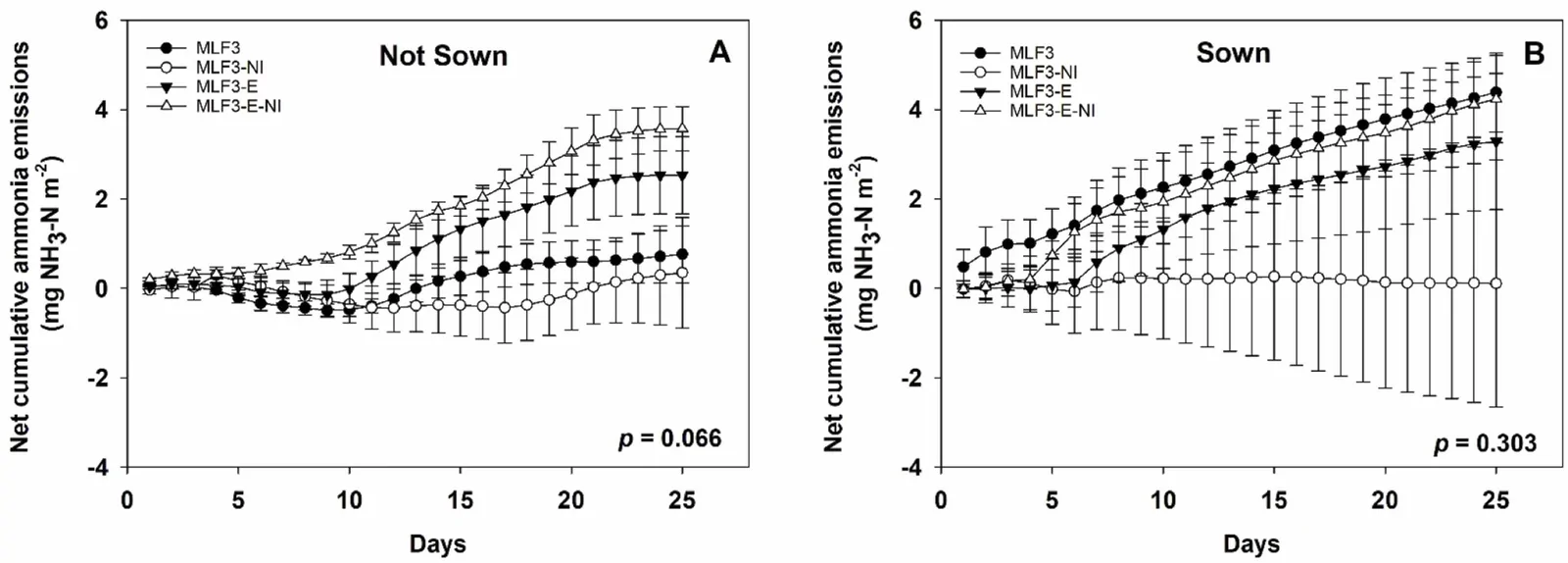
Net cumulative ammonia emission from soils not-sown (A) or sown (B) with ryegrass, over the monitoring period of 25-days. Each datapoint represents the mean value of four replicates after correcting for emissions from the Control treatment. MLF3 and MLF3-NI are the original multilayer filters without or with the nitrification inhibitor dicyandiamide, respectively; MLF3-E and MLF3-E-NI are multilayer filters, enriched with emitted gases from pig slurry, without or with nitrification inhibitor, respectively.

Net cumulative NH_3_ emissions were generally slightly higher in not-sown treatments than the equivalent sown treatments (Fig. 4), ranging from ∼0–4 mg NH_3_-N m^−2^ in 25 days. No statistical differences were observed between treatments with respect to cumulative NH_3_-N values. However, it should be highlighted that the emission pattern was different in not-sown and sown treatments due to enriched MLF (overall with NI) emitted more NH_3_-N than other treatments in the not-sown soil, in contrast to what happened in sown soils, where MLF3 and MLF3-E-NI produced the greatest emissions (Fig. 4).

Net cumulative CO_2_ emissions were significantly greater in not-sown treatments than in sown ones (*p* = < 0.001), whilst N_2_O showed the opposite pattern (Figure S9A and S9B, respectively). CO_2_ emissions from the Control were ∼6 fold lower than in amended soils (data not shown) but no differences were observed between MLF treatments (Figure S9A). Cumulative N_2_O emissions were significantly greater from the Control treatment relative to the MLFs, in which no emissions were detected in not-sown soils. Eventually, no significant differences were observed between MLF treatments, with a large amount of variance in cumulative N_2_O emissions from each treatment (Fig S9B).

### 3.5 Leached nitrogen from soil

**Fig. 5:**
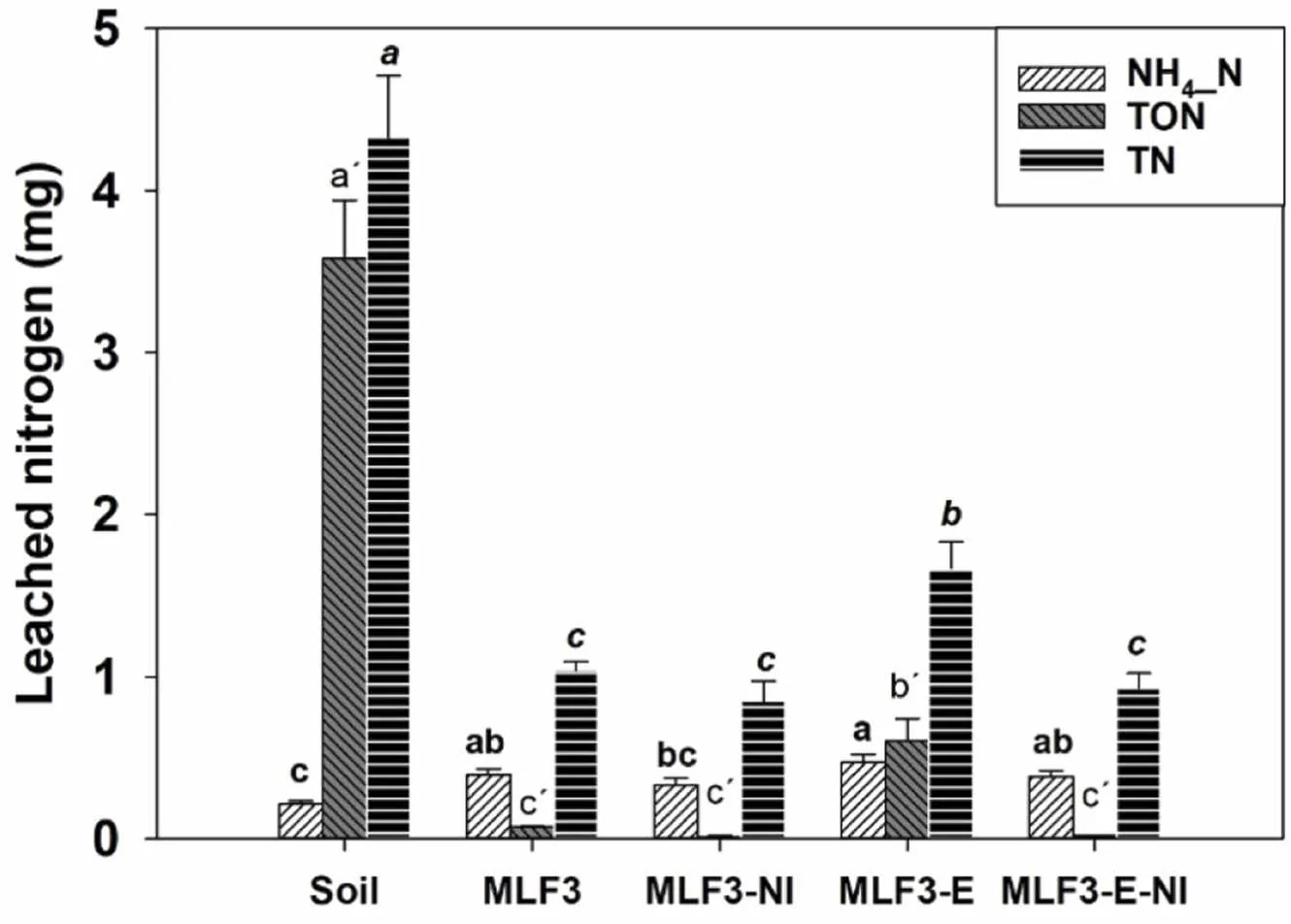
Soluble nitrogen forms leached from not-sown soil fertilized with MLF3 (original or enriched), with or without the nitrification inhibitor dicyandiamide. Columns are mean values ± SE (*n* = 4). Different letters represent statistically significant differences between treatments.

The different forms of soluble N were only analyzed in not-sown soils at the end of the experiment. The Control treatment had the greatest amount of N, mainly in total oxidized N (TON) form, followed by MLF3-E in the leachate (Fig. 5). The latter produced the greatest concentration of NH_4_-N in the leachate, significantly higher than MLF3-NI and Control treatments (Fig. 5). TON was statistically greater in leachates from the Control treatment than in those from MLF3-E, which in turn was greater than other treatments, occurring the same for TN.

## 4 Discussion

### 4.1 Gas measurement and data analysis approach

A key methodological aspect of this study that deserves to be discussed is the comparison between area under the curve (AUC) and linear regression (LR) approaches for flux estimation. Although both methods showed consistent relative trends between treatments, absolute values differed by approximately one order of magnitude. This discrepancy is mainly related to the assumptions underlying each method. The LR approach assumes a linear increase in gas concentration during chamber closure, which may not hold for all gases, particularly under low flux conditions or when concentration dynamics are non-linear. In contrast, the AUC method integrates concentration over time and is less dependent on linearity. In our system, the LR method showed a total of 25 mg of total N volatilized compared to the ∼2.5 mg of AUC. Therefore, LR was more consistent with the real N mass balance (∼110 mg), suggesting a higher accuracy for cumulative flux quantification when linearity criteria are met. However, the AUC method proved useful for capturing relative differences among treatments, especially for gases with high variability such as CH_4_ and N_2_O. This is because AUC does not rely on assumptions of linear concentration increase, making it more robust under variable emission conditions but potentially less accurate for absolute flux quantification. Even though lesser number of studies used AUC method for flux calculations (e.g.,Cardenas et al., 2022), we considered very useful in a multi gas study like this. Therefore, both methods should be interpreted as complementary: LR for quantitative flux estimation and AUC for comparative trend analysis, mainly.

### 4.2 Gas mitigation performance of multilayer filters

This study confirms that MLFs can effectively capture and reduce gaseous emissions from pig slurry, with total N capture rates between 76–98% and significant reductions in cumulative NH_3_ and NO_2_ emissions. However, filters had a greater N-enrichment in MLF1 >MLF2 >MLF3, whilst NH_3_ and NO_2_ gas capture efficacy showed opposite pattern, either calculated with AUC or LR method. This indicates that other volatile forms of N are being captured, but it is also possible that the N captured in MLF2 and MLF3 is lost at a higher rate compared to MLF1 between end of experiment and analyzing time. On the other hand, the mass balance deviation among total N volatilized and the sum of all N forms may be due to the N-gap caused by full denitrification to N_2_ which was neither measured nor estimated (Neysari et al., 2023). The deviation between AUC and LR fluxes calculation could depend on the parameters chosen, such as delaying time or determination coefficient threshold for LR (in this case 0.6) and other causes commented in the previous section. In any case, the relative values between calculation methods with respect to the Control were similar, which assured the efficacy performance of the filters. MLF3 achieved the highest capture efficacy of NH_3_ and NO_2_ (Fig. 1 ans S5). This is most likely due to the acid pH and increased specific surface area associated with the filter materials (orange peel) and the enhanced photocatalytic properties (titanium and zinc oxides contained in the Al sludge) that transform NO_2_ to NO_3_ in the presence of light in a humid environment (Table S1; Balbuena et al., 2015; Barrón et al., 2020). The greater efficacy of MLF2 relative to MLF1 is probably caused by the greater filter mass content of orange peel and the inclusion of cork and bentonitic sands, which increase the specific surface area (Table 3). Lastly, MLF2 contained a greater Compost EM content, which is known to contain ligands that can capture NH_3_ (Van der Heyden et al., 2015). These efficacy results are comparable or superior to conventional biofiltration systems such as woodchip, zeolite and/or compost-based (Chen and Hoff, 2009, Van der Heyden et al., 2015), but the methodology and technology differ. For example, we use a multilayer system —without mixing materials— that showed synergy effects (González-Guzmán et al., 2026), and we also combine organic and inorganic materials in our study. We did not define a gas flow through the filter, instead, gases passed through the MLFs by diffusion. Thus, it was not possible to calculate the empty bed residence time (EBRT, the theoretical time that the gases remain within the multilayer filter bed), necessary when we scale up this technology to calculate the capture modelling approach.

The Control and MLF3 had the lowest cumulative CO_2_ emission values. This result was unexpected as Olive ashes (present in MLF1) and black slag (present in MLF2) have demonstrated efficient CO_2_ capture capacity by carbonatation in other experiments (González-Guzmán et al., 2026).The ineffective capture of CO_2_ found in this study is possibly caused by an insufficient mass of olive ashes and black slag in the MLFs. This should not have favored the carbonatation process, but it could be linked to an additional CO_2_ source like microbial activity (respiration) within the filter layers (L. Chen and Hoff, 2009; Swanson and Loehr, 1997). However, we did observe C enrichment in all MLF treatments (MLF1 >MLF2 >MLF3), suggesting that MLFs retained volatile C compounds emitted from slurry, which were partially biodegraded by the microbial community present in the MLFs. Thus, resulting in an additional CO_2_ source and a final net accumulation of total C in the filter material (Boswell, 2004, Colón et al., 2009).

Despite the high capture efficacy of NH_3_ and NO_2_ observed in this study, the MLFs did not consistently reduce all emitted gases including N_2_O and CH_4_ (supplementary material, Figure S7 and S8). Therefore, we conclude that our hypothesis was partially achieved, we were unable to reach the minimum 50% mentioned for all gases such as CO_2_. Further work is needed to find and assess residue formulations for the most optimal gas capture. Additionally, the experiments presented here need to be scaled up to better relate our findings to real-world systems, to test the MLFs under greater gas emissions sources and to improve the data used to generate gas flux curves (Rochette and Eriksen-Hamel, 2008). In this sense, we already found some limitations for installing the technology in the field. Technologically, the flux of the gas must be controlled to enhance the efficacy of the filter and to also adjust it to different environmental conditions. This is a key point because it will also allow better separation of the filter matrix from the slurry, which will enhance the operability for the farmer and decrease the likelihood of enriching the filter with fecal bacteria. On the other hand, this technology and circular economy strategy warrants further investigation to ensure it achieves the techno-scientific reports evidencing lack of risk either for crops or human health, whilst also being environmentally and economic viable, due to the costs associated with waste transport.

### 4.3 Fertilizer potential of MLF3 as a soil amendment

MLF3 complies with EU Regulation 1009/2019 (CFP 3b) as an inorganic amendment derived from recycled material. Physical and chemical fertility of the Dystric Cambisol used in Experiment 2 was high, as it has a balance texture, moderate levels of N, moderate-high levels of Ca, K and P. From an agronomic point of view, this soil could be enhanced by increasing the pH to a value of 6.5 (to avoid Al availability) and the CEC to a value of 12–14 cmol^+^ kg^−1^. Therefore, the amendment enhanced the levels of OM, CEC, and N, mainly, but had not the optimum pH for this specific soil and crop. Even though, this amendment increased the germination rate of *Lolium perenne* in all treatments (Fig. 2) relative to the Control and was linked to a reduced ADM yield. We suggest that the reduced ADM observed under the MLF3 amended soil was caused by soil volume restriction within the pots used and competition between plants, leading to higher RDM/ADM ratios. Under competitive conditions plants prioritize growth of root biomass to secure nutrient supply (Berendse and Möller, 2009). Authors discard that ADM decrease was because toxicological effects due to the MLF3 material, which was selected for the agronomic experiment, showed concentrations below EU Regulation 2019/1009 for PFC 3(B) inorganic soil improvers, including Cd, Ni, Pb, As, Cu and Zn (1.5, 100, 120, 40, 300 and 800 mg kg^−1^, respectively). However, this comparison should be interpreted with caution because the regulation establishes a specific limit for Cr(VI), whereas only total Cr was determined in this study. Therefore, Cr speciation, together with any additional regulated parameters not quantified here (inorganic As), should be included in future validation steps before full regulatory compliance can be claimed.

The factors “enriched” and “without NI” were associated with enhanced ryegrass yield, which is related with the greater N uptake by ryegrass under MLF3-E and MLF3-E-NI compared to the original filter. A critical and essential aspect of this study was to capture the nutrients which would otherwise be lost from slurry tanks to the atmosphere or through leaching and make them available for crops. Our findings therefore support the fertilizer potential of this amendment and highlight the potential benefits of a further development of this technology.

Surprisingly, we observed increased S uptake by ryegrass under the original MLF treatments, relative to MLF-E. This paradox may be explained by differences in S speciation during filtration and in soil. During the filtration process S captured from VSC ((Rumsey et al., 2014)) could be retained in recalcitrant forms such as elemental S, metal sulfides, or organically bound S (Edwards, 1998, Malik et al., 2021) rather than as readily available sulfate. In addition, the higher C and N availability in MLF-E may have promoted microbial immobilization (Nziguheba et al., 2005), further limiting availability of S. In this case, using the MLF3 as amendment fulfilled our hypothesis that the nutrients enriched in the filter were able to increase biomass and/or nutrients uptake. However, it is true that no differences were observed respect to Control in terms of TDM.

Eventually, even though MLF1 was not valorized as amendment, EU Regulation 2019/1009 PFC 3(B) for inorganic soil improvers limits the Cu ≤ 300 mg kg⁻¹ and Pb ≤ 120 mg kg⁻¹ , which was exceeded by MLF1, Cu by 70% and Pb by more than 5-fold. This is a major regulatory concern that must be taken into account for the formulation of filters and soil amendments.

### 4.4 Post-application emissions and water-soluble nutrients

Soil gas emissions (NH_3_ and N_2_O) were slightly higher in sown than not-sown soils except for CO_2_, due to CO_2_ uptake during photosynthesis under daylight conditions. Ammonia emissions generally increased in not-sown soil amended with MLFs-E, especially when combined with NI. This is because of the higher content of N (mainly as NH_4_^+^) and the inhibition of the nitrification process, which lead to a higher loss of NH_3_-N (Wu et al., 2021). The opposite occurs when the soil is sown (except for MLF-NI), which supports our hypothesis that the N gained in slurry tanks can be made available for plant uptake, rather than being lost to the atmosphere and that microbial competition for NH_3_ can mitigate emissions (Kuzyakov and Xu, 2013). This finding should be considered in future work to align the time of fertilization and sowing to avoid the loss of nutrients to the atmosphere. N_2_O emissions were lower in the amended not-sown soils, which is key for two main reasons: first, the amendment seemingly prevents the emission of potent greenhouse gas such as N_2_O; and second, it enables the immobilization of nitrates (precursor forms of N_2_O). Therefore, N in the amended not-sown-soil remains available for a longer period while preventing leaching. These results are closely related to the significantly greater concentration of oxidized N forms (TON, such as nitrates) and TN in the leachate found in control treatment. Leachate analysis showed reduced soluble-N availability in MLF-amended soils, particularly in terms of TON and TN. This analysis also showed functionality of the NI DCD preventing nitrification and therefore the loss of N. This is mainly seen in MLF3-E treatment, which showed a significant increase in TON forms in the leachate (after Control) because it was enriched and without NI.

### 4.5 Residue valorization and circular economy innovation

This study demonstrates the feasibility of upcycling a selection of heterogeneous waste-based materials via an agro-environmental technology, reinforcing the potential of waste-based materials to align with circular economy principles (Chojnacka et al., 2020, Commission, 2020). This system exemplifies how cross-sectoral residue integration —agriculture, food industry, metal processing— can lead to multifunctional, sustainable solutions. Thus, waste valorization could complement or replace costly air-scrubber systems, promoting wider adoption of air pollution control technologies, because of its modular and low-tech nature which suggests high scalability. This study is an example of how increasing knowledge of how to use waste-based materials within circular economy aims to benefit society and the environment, integrating these types of alternatives into policy frameworks, for example as a Best Available Practice. This technology can recycle waste materials and enhance soil properties, whilst playing a critical role in minimizing pollutant emissions (Ravindran et al., 2021, Walling and Vaneeckhaute, 2020). Nevertheless, from an environmental perspective, the reuse of waste-derived materials as soil amendments requires careful consideration of potential risks associated with trace element accumulation. Although the total concentrations of some elements (such as Cu and Pb) in the MLFs were relatively high, their plant-available fractions were low (Table 3), suggesting limited short-term availability. However, it is true that long-term application and repeated use could lead to the gradual accumulation of certain elements in soil, which may pose environmental or agronomic risks. Therefore, further studies assessing long-term effects, including metal mobility and plant uptake under field conditions, are recommended to ensure the safe implementation of this technology.

## 5 Conclusions

The results demonstrate that residue-based multilayer filters can effectively capture gaseous emissions from pig slurry, particularly NH_3_ and NO_2_ (from 51% to 76% and from 37% to 95%, respectively). In contrast, mitigation of CO_2_ was inconsistent between MLFs (from −37–48%). CH_4_ and N_2_O emissions were generally low, showing a higher variation along the experiment, being the capture efficacy moderate (∼40%) and almost null, respectively. These MLFs can also be used as a nutrient enriched amendment (N, C and S) within the experimental setup applied in this study. This novel dual functionality supports their potential use within circular economy strategies, to close nutrient cycles within livestock systems, minimizing both environmental losses and mineral fertilizer inputs. In addition, the reuse of enriched filters as soil amendments showed potential for nutrient recycling and plant uptake, although effects on total dry matter were not significant. The observed NH_3_ losses from enriched filters in not-sown soil suggests the importance of the crop for retaining and using N from pig slurry, meaning that incubation time is very relevant. Eventually, these observations highlight the importance of the agronomic context —plant presence, soil type, and nitrification inhibitor use— in maximizing the environmental benefits of the filter residues, with the formulation process being key to efficient gas capture and to minimize environmental impacts of livestock farming, such as GHGs emission, leaching or accumulation of potential toxic elements in the edible part of the crop.

Overall, this study provides a proof-of-concept for integrating gas capture and nutrient recovery using waste-derived materials, but further research is required to optimize filter formulation, assess long-term environmental risks, and validate performance and economic viability under real farming conditions, which is essential to achieve end-of-waste status for some materials.

## CRediT authorship contribution statement

**Vidal Barrón:** Writing – original draft, Supervision, Resources, Methodology, Investigation, Conceptualization. **Gonzalez Guzmán Adrián:** Writing – original draft, Visualization, Supervision, Resources, Project administration, Methodology, Investigation, Funding acquisition, Formal analysis, Data curation, Conceptualization. **Alison Carswell:** Writing – review & editing, Writing – original draft, Validation, Supervision, Investigation, Funding acquisition, Conceptualization. **Sanchez-Rodriguez Antonio Rafael:** Writing – original draft, Visualization, Supervision, Investigation. **Nadine Loick:** Writing – original draft, Supervision, Methodology, Investigation, Formal analysis, Data curation.

## Declaration of Generative AI and AI-assisted technologies in the writing process

Adrian thanks ChatGPT4o for helping him to improve English language of the first draft of the manuscript, and also for correcting the code needed in the software R to perform either analysis or figures.

## Declaration of Competing Interest

The authors declare that they have no known competing financial interests or personal relationships that could have appeared to influence the work reported in this paper.

## Data Availability

Data will be made available on request.
