# Supplementary material for: Residue-based multilayer-filters reused as amendment after capturing pig slurry gases
Source: Environ Technol Innov. 2026 Sep;43:105111. doi: 10.1016/j.eti.2026.105111 (PMC13576387; doi:10.1016/j.eti.2026.105111)
Supplement: Supplementary file 1 [file mmc1.docx]

**SUPLEMENTARY MATERIAL**

**Figure S1.** Mean, maximum and minimum temperature and relative humidity mean evolution during Experiment 1.

**A**

**B**

**C**

**D**

**E**

**F**

**G**

**Figure S2.** Visual representation of the methodology used either in Experiment 1 (A, B and C) or Experiment 2 (D, E, F and G). Figure 1A shows the representation of the twelve experimental units, 1B shows one experimental unit in more detail and 1C the coupled gas analyzers. Figure 1D, 1E and 1F show the dynamic air throughflow system used with acid traps fitted at outlets, which captured NH_3_ emitted from the amended soil. Pots were placed inside the sealed orange chambers during the simulated nighttime period (according to the method of Misselbrook et al. (2005) as adapted by Drame et al. (2023)). Figure 1F shows the automatic chambers used to measure the GHGs emissions from soil amended during the day using a Gasmet measurement system (Gasmet Technologies (UK) Ltd).

**Figure S3.** Scheme of the system built to measure all gases that pass through the multilayer filters in Experiment 1. The upper image represents the scheme just for one automatic chamber, whilst the image below shows the 12 automatic chambers connected to the multiplex, which gives the orders to open/close purge gases from automatic chambers.

**Figure S4.** Total N of the fresh liquid slurry (LS), and N distribution between dry matter slurry (DMS), volatilized N (Volatilized) and captured N for each multilayer filter treatment (Captured). MLF1, 2 and 3 are multilayer filters. Bars are mean values ± SE (n= 3).

| **Table S1.** Physical and chemical properties of all residues that form the multilayer filters (MLFs) | | | | | | | | | | | | | | |
| --- | --- | --- | --- | --- | --- | --- | --- | --- | --- | --- | --- | --- | --- | --- |
| **Descripcion** | **Particle size** | | | | **Specefic Surface** | **Density** | **pH_H20_** | **EC** | **CaCO_3_** | **CECe** | **Ca_CEC_** | **Mg_CEC_** | **Na_CEC_** | **K_CEC_** |
|  | **2-0.5** | **0.5-0.2** | **0.2-0.05** | **<0.05** |  |  |  |  |  |  |  |  |  |  |
|  | **mm** | | | | **m^2^ g^-1^** | **g cm^-3^** |  | **µS cm^-1^** | **%** | **cmolc kg^-1^** | | | | |
| Al chips | 44 | 22 | 28 | 6 | 86 | 1.21 | 9.1 | 1056 | 6 | 57 | 16 | 24 | 13 | 5 |
| Al sludge | 62 | 19 | 16 | 3 | 352 | 0.56 | 8.2 | 5620 | 5 | 136 | 47 | 2 | 85 | 2 |
| Bentonitic sands | 15 | 5 | 59 | 21 | 153 | 0.92 | 10.0 | 1526 | 5 | 136 | 43 | 3 | 88 | 1 |
| Black slag biomass | 73 | 19 | 8 | 0 | 423 | 0.79 | 12.8 | 1244 | 15 | 169 | 34 | 3 | 8 | 125 |
| Compost EM | 5 | 37 | 46 | 12 | 222 | 0.77 | 6.0 | 12640 | 0 | 265 | 223 | 17 | 3 | 22 |
| Cork | 51 | 21 | 22 | 7 | 369 | 0.49 | 6.8 | 476 | 0 | 87 | 75 | 6 | 1 | 6 |
| Iron chips | 2 | 20 | 60 | 18 | 5 | 0.36 | 5.5 | 450 | 0 | 3 | 2 | 0 | 1 | 1 |
| Iron slag | 66 | 18 | 11 | 5 | 9 | 2.13 | 10.8 | 821 | 15 | 362 | 302 | 56 | 4 | 0 |
| Olive ash Baena | 20 | 13 | 41 | 27 | 57 | 0.71 | 13.2 | 11310 | 34 | 1594 | 1037 | 2 | 249 | 306 |
| Orange peel | 10 | 21 | 60 | 9 | 603 |  |  | 4630 | 0 | 24 | 10 | 3 | 0 | 12 |
| Sludge M | 4 | 38 | 45 | 13 | 232 | 0.77 | 6.5 | 11080 | 0 | 221 | 204 | 10 | 1 | 6 |
| UBCEP^2^ | 100 | 0 | 0 | 0 | 345 | 0.09 | 9.9 | 9370 | 2 | 77 | 5 | 1 | 69 | 3 |
| ^1^Al: Aluminum; ^2^UBCEP: Uncooked biomass from the cellulose extraction process; | | | | | | | |  |  |  |  |  |  |  |

| **Table S1 (continued).** Physical and chemical properties of all residues that form the multilayer filters (MLFs) | | | | | | | | | | | | |
| --- | --- | --- | --- | --- | --- | --- | --- | --- | --- | --- | --- | --- |
| **Descripcion** | **Carbon forms** | | | **Nitrogen forms** | | | **S_T_** | **P_olsen_** | **DTPA** | | | |
|  | **C_T_** | **C_org_** | **C_piro_** | **N_T_** | **NO_3_** | **NH_4_** |  |  | **Fe_DTPA_** | **Cu_DTPA_** | **Mn_DTPA_** | **Zn_DTPA_** |
|  | **g kg^-1^** | | | **mg kg^-1^** | | | **%** | **mg kg^-1^** | | | | |
| Al chips |  |  |  | 3138 | 66 | 81 | 0.02 | 8 | 98 | 279 | 27 | 206 |
| Al sludge | 10 | 4 |  | 405 | 84 | 5 | 0.96 | 0 | 55 | 10 | 3 | 143 |
| Bentonitic sands | 178 |  |  | 2212 | 3 | 87 | 0.20 | 1 | 102 | 14 | 26 | 53 |
| Black slag biomass |  |  |  | 568 | 33 | 2 | 0.03 | 368 | 37 | 21 | 75 | 9 |
| Compost EM | 156 | 173 |  | 19730 | 16235 | 167 | 0.47 | 103 | 360 | 29 | 187 | 103 |
| Cork | 374 | 244 |  | 8125 | 203 | 45 | 0.12 | 58 | 118 | 14 | 199 | 9 |
| Iron chips |  |  |  | 124 | 168 | 7 |  | 2 | 705 | 337 | 245 | 25 |
| Iron slag |  |  |  | 1377 | 71 | 5 | 4.72 | 0 | 1 | 3 | 1285 | 2 |
| Olive ash Baena | 99 | 19 |  | 356 | 1677 | 10 |  | 2660 | 1154 | 140 | 5 | 4 |
| Orange peel | 436 | 337 |  | 11551 | 165 | 9 | 0.22 | 83 | 18 | 13 | 3 | 5 |
| Sludge M | 131 | 141 |  | 14280 | 16507 | 256 | 0.46 | 236 | 353 | 38 | 81 | 95 |
| UBCEP^2^ | 488 | 470 | 31 | 671 | 516 | 25 |  | 44 | 12 | 13 | 32 | 3 |
| ^1^Al: Aluminum; ^2^UBCEP: Uncooked biomass from the cellulose extraction process; | | | | | | | | | | | | |

| **Table S1 (continued).** Physical and chemical properties of all residues that form the multilayer filters (MLFs) | | | | | | | | | | | | | | | | | | | | | | | | | | |
| --- | --- | --- | --- | --- | --- | --- | --- | --- | --- | --- | --- | --- | --- | --- | --- | --- | --- | --- | --- | --- | --- | --- | --- | --- | --- | --- |
| **Descripcion** |  | | | | | | | | | | | | | | | | | | | | | | | | | |
|  | **Al_T_** | **As_T_** | **B_T_** | **Ba_T_** | **Ca_T_** | **Cd_T_** | **Cl_T_** | **Co_T_** | **Cr_T_** | **Cu_T_** | **Fe_T_** | **K_T_** | **Mg_T_** | **Mn_T_** | **Mo_T_** | **Na_T_** | **Ni_T_** | **PT** | **Pb_T_** | **S_T_** | **Sb_T_** | **Si_T_** | **Sn_T_** | **Sr_T_** | **Ti_T_** | **Zn_T_** |
|  |  | | | | | | | | | | | | | | | | | | | | | | | | | |
| Al chips | > 80000 | 1.8 | 50 | 2550 | 8800 | 2.9 | 7351 | 9.2 | 78 | 2130 | 43500 | 3300 | 10700 | 418 | 17.7 | 4230 | 309.0 | 520 | 5000.0 | 10712 | 167.0 | 46811 | 538.0 | 458 | 7500 | 2410 |
| Al sludge | > 80000 | 37.3 | 271 | 160 | >250000 | 0.3 | 398 | 3.4 | 310 | 22 | 5100 | 3000 | 3700 | 58 | 11.9 | 15700 | 173.0 | 330 | 5.9 | 9889 | 0.6 | 2403 | 108.0 | 93 | 3100 | 45 |
| Bentonitic sands | 43800 | 2.3 | 37 | 265 | 15300 | 0.1 | 716 | 3.0 | 10 | 20 | 18200 | 1200 | 12400 | 560 | 0.7 | 12000 | 3.8 | 220 | 14.3 | 2015 | 0.2 | 158807 | 2.0 | 94 | 500 | 225 |
| Black slag biomass | 15858 | 4.6 | 60 | 172 | 72439 |  | 5299 | 3.0 | 31 | 122 | 10366 | 46123 | 8927 | 1154 |  | 4179 | 30.5 | 7528 | 6.1 | 5842 |  | 61900 |  | 290 | 1398 | 68 |
| Compost EM | 13200 | 9.7 | 27 | 54 | 28800 | 0.5 |  | 5.8 | 52 | 129 | 16900 | 3700 | 3900 | 580 | 2.2 | 660 | 19.8 | 10200 | 54.4 |  | 2.0 |  | 9.6 | 82 | <100 | 347 |
| Cork | 5410 |  | 1 | 69 | 18365 |  | 1176 | 0.1 | 10 | 11 | 4856 | 1395 | 4207 | 353 |  | 194 | 1.7 | 258 | 3.2 | 1293 |  | 27881 |  | 75 | 648 | 30 |
| Iron chips | 315 | 43.3 | 12 | 3 | 1146 |  |  | 76.5 | 6388 | 820 | 467073 | 734 | 146 | 10244 | 175.7 | 1131 | 2741.3 | 315 | 6.3 | 354 |  | 4421 | 17.5 |  |  | 200 |
| Iron slag | 4700 | 11.6 | 3 | 9 | 71700 | < 0,01 | 154 | 5.3 | 95 | 567 | 139000 | 200 | >100000 | 7380 | 6.5 | 340 | 32.8 | 50 | 9.7 | 73070 | 0.8 | 37450 | 13.3 | 108 | 100 | 200 |
| Olive ash Baena | 5167 | <5.000 | 411 | 98 | 284446 | <1.000 | 35033 | <5.000 | 20 | 195 | 6175 | 81555 | 22796 | 398 | 2.8 | 34032 | 13.4 | 7631 | 4.1 | 4652 | <10.00 | 6203 | <5.0 | 878 | 271 | <50.0 |
| Orange peel | 6 |  | 18 | 2 | 5050 |  |  |  |  | 4 | 697 | 8488 | 1468 | 8 |  | 251 |  | 909 |  |  |  |  |  | 26 |  | 9 |
| Sludge M | 17000 | 10.8 | 24 | 58 | 30100 | 3.7 |  | 6.5 | 84 | 148 | 19600 | 2500 | 3700 | 681 | 2.7 | 320 | 24.3 | 11200 | 70.4 |  | 1.6 |  | 13.7 | 86 | 100 | 411 |
| UBCEP^2^ | <100 | 0.7 | 6 | 12 | 1600 | < 0.01 | 218 | < 0.1 | 2 | 1 | <100 | 600 | 200 | 40 | 0.1 | 10300 | 0.3 | 110 | 0.3 | 3380 | < 0.02 | 2846 | < 0.05 | 7 | <100 | < 0.1 |

| Table S2. Quality analysis and control parameters from the different labs for the different elements | | | | | | | | | | | |
| --- | --- | --- | --- | --- | --- | --- | --- | --- | --- | --- | --- |
| **Analyte Symbol** | **ActLabs** | | | **AGQ** | | | | **SCAI** | | | |
|  | **Unit Symbol** | **LOQ** | **Analysis Method** | **Unit Symbol** | **LOQ_min** | **LOQ_max** | **Analysis Method** | **LOD** | | **LOQ** | |
| Ag | ug/L | 5 | ICP-OES | mg/Kg | 0.5 | 10000 | ICP-MS | µg/L |  | mg/L |  |
| Al | mg/L | 0.1 | ICP-OES |  |  |  |  | µg/L |  | mg/L |  |
| As | ug/L | 30 | ICP-OES | mg/Kg | 5 | 10000 | ICP-MS | µg/L | 0.5 | mg/L | 0 |
| Ba | ug/L | 20 | ICP-OES |  |  |  |  | µg/L |  | mg/L |  |
| Be | ug/L | 2 | ICP-OES |  |  |  |  | µg/L |  | mg/L |  |
| Bi | ug/L | 20 | ICP-OES |  |  |  |  | µg/L |  | mg/L |  |
| Ca | mg/L | 0.1 | ICP-OES |  |  |  |  | µg/L |  | mg/L |  |
| Cd | ug/L | 2 | ICP-OES | mg/Kg | 0.1 | 100 | ICP-MS | µg/L | 0.5 | mg/L | 0.5 |
| Ce | ug/L | 30 | ICP-OES | mg/Kg | 0 | 0 | ICP-MS | µg/L |  | mg/L |  |
| Co | ug/L | 2 | ICP-OES | mg/Kg | 5 | 10000 | ICP-MS | µg/L | 1 | mg/L | 0.5 |
| Cr | ug/L | 20 | ICP-OES | mg/Kg | 1 | 100 | ICP-MS | µg/L | 0.5 | mg/L | 1 |
| Cu | ug/L | 2 | ICP-OES |  |  |  |  | µg/L |  | mg/L |  |
| Fe | mg/L | 0.01 | ICP-OES |  |  |  |  | µg/L |  | mg/L |  |
| K | mg/L | 0.1 | ICP-OES |  |  |  |  | µg/L |  | mg/L |  |
| Li | mg/L | 0.05 | ICP-OES | mg/Kg | 5 | 10000 | ICP-MS | µg/L |  | mg/L |  |
| Mg | mg/L | 0.1 | ICP-OES |  |  |  |  | µg/L |  | mg/L |  |
| Mn | mg/L | 0.01 | ICP-OES |  |  |  |  | µg/L |  | mg/L |  |
| Mo | ug/L | 5 | ICP-OES | mg/Kg | 1 | 10000 | ICP-MS | µg/L | 1 | mg/L | 0.5 |
| Na | mg/L | 0.1 | ICP-OES |  |  |  |  | µg/L |  | mg/L |  |
| Ni | ug/L | 5 | ICP-OES | mg/Kg | 1 | 100 | ICP-MS | µg/L | 1 | mg/L | 1 |
| P | mg/L | 0.02 | ICP-OES |  |  |  |  | µg/L |  | mg/L |  |
| Pb | ug/L | 10 | ICP-OES | mg/Kg | 0.5 | 10000 | ICP-MS | µg/L | 1 | mg/L | 1 |
| S | mg/L | 1 | ICP-OES |  |  |  |  | µg/L |  | mg/L |  |
| Sb | ug/L | 10 | ICP-OES | mg/Kg | 1 | 10000 | ICP-MS | µg/L | 1 | mg/L | 1 |
| Se | ug/L | 20 | ICP-OES | mg/Kg | 5 | 10000 | ICP-MS | µg/L |  | mg/L |  |
| Si | mg/L | 0.1 | ICP-OES |  |  |  |  | µg/L |  | mg/L |  |
| Sn | ug/L | 10 | ICP-OES | mg/Kg | 5 | 10000 | ICP-MS | µg/L | 1 | mg/L | 1 |
| Sr | ug/L | 10 | ICP-OES |  |  |  |  | µg/L |  | mg/L |  |
| Te | ug/L | 10 | ICP-OES |  |  |  |  | µg/L |  | mg/L |  |
| Ti | ug/L | 10 | ICP-OES |  |  |  |  | µg/L |  | mg/L |  |
| Tl | ug/L | 10 | ICP-OES | mg/Kg | 1 | 10000 | ICP-MS | µg/L |  | mg/L |  |
| U | mg/L | 0.05 | ICP-OES |  |  |  |  | µg/L |  | mg/L |  |
| V | ug/L | 10 | ICP-OES | mg/Kg | 5 | 10000 | ICP-MS | µg/L |  | mg/L |  |
| W | ug/L | 10 | ICP-OES |  |  |  |  | µg/L |  | mg/L |  |
| Y | ug/L | 10 | ICP-OES |  |  |  |  | µg/L |  | mg/L |  |
| Zn | ug/L | 5 | ICP-OES |  |  |  |  | µg/L |  | mg/L |  |

**Figure S5.** Capture efficacy (%) of NO_2_-N, NH_3_-N and CO_2_-C gases for each of the three multilayer filters (MLF) relative to the Control treatment. Data obtained area under the curve (AUC) method. Different letters between treatments indicate significant differences (*p* < 0.05) after one-way ANOVA (*n* = 3). Absence of letters means no statistical differences.

**Figure S6.** Capture efficacy (%) of N_2_O-N and CH_4_-C gases for each multilayer filter (MLF) relative to the Control, calculated using area under the curve (AUC; panels A, B and C) and linear regression (LR; panels D, E and F) methods. Different letters between treatments indicate significant differences (*p* < 0.05) after one-way ANOVA (*n* = 3). Absence of letters means no statistical differences.

**Figure S7.** Daily cumulative emissions (mg m^-2^) of NO_2_-N, NH_3_-N and CO_2_-C gases for each multilayer filter (MLF), calculated using area under the curve (AUC; panels A, B and C) and linear regression (LR; panels D, E and F) calculation methods. Different letters mean statistical differences among treatments.

.

**Figure S8.** Daily cumulative emissions (mg m^-2^) N_2_O-N and CH_4_-C gases for each multilayer filter (MLF), calculated using area under the curve (AUC; panels A and B) and linear regression (LR; panels C and D) calculation methods. Different letters mean statistical differences among treatments.

|  |  |
| --- | --- |
| **Figure S9.** Net cumulative CO_2_ (A) and N_2_O (B) emissions (after subtracting the Control emissions) in each of the four MLF treatments for 25 days monitoring (MLF3 and MLF3-NI are the original multilayer filters without or with nitrification inhibitor, respectively; MLF3-E and MLF3-E-NI are enriched multilayer filter without or with nitrification inhibitor, respectively) either for not-sown (white bars) and sown (black bars) soils. Bars are mean values ± SE (*n* = 4). Standard errors are not shown for N_2_O emissions in unsown soils because no emissions were detected in the MLF treatments | |
